# Familial Longevity Is Marked by Better Cognitive Performance at Middle Age: The Leiden Longevity Study

**DOI:** 10.1371/journal.pone.0057962

**Published:** 2013-03-04

**Authors:** Marjon Stijntjes, Anton J. M. de Craen, Diana van Heemst, Carel G. M. Meskers, Mark A. van Buchem, Rudi G. J. Westendorp, P. Eline Slagboom, Andrea B. Maier

**Affiliations:** 1 Department of Gerontology and Geriatrics, Leiden University Medical Center, Leiden, The Netherlands; 2 Department of Internal Medicine, Section of Gerontology and Geriatrics, VU University Medical Center, Amsterdam, The Netherlands; 3 Netherlands Consortium for Healthy Ageing, Leiden, The Netherlands; 4 Department of Rehabilitation Medicine, Leiden University Medical Center, Leiden, The Netherlands; 5 Department of Radiology, Leiden University Medical Center, Leiden, The Netherlands; 6 Leyden Academy on Vitality and Ageing, Leiden, The Netherlands; 7 Department of Medical Statistics, Section of Molecular Epidemiology, Leiden University Medical Center, Leiden, The Netherlands; INRCA, Italy

## Abstract

**Background:**

Decline in cognitive performance is a highly prevalent health condition in elderly. We studied whether offspring of nonagenarian siblings with a familial history of longevity, perform better on cognitive tests compared to their partners as controls. This is relevant since it could provide insights into determinants underlying decline in cognitive performance.

**Methods:**

Cross-sectional analysis within the longitudinal cohort of the Leiden Longevity Study consisting of middle-aged offspring of nonagenarian siblings together with their partners (n = 500, mean age (SD) 66.3 (6.1) and 65.7 (7.2) years, respectively) as controls. Memory function, attention and processing speed were tested using the 15-Picture Learning Test, Stroop test and Digit Symbol Substitution Test. Data were analyzed with regression adjusted for age, gender, years of education and additionally for diabetes mellitus, cardiovascular diseases, alcohol use, smoking, inflammatory markers and *apolipoprotein E* genotype. Robust standard errors were used to account for familial relationships among the offspring.

**Results:**

Cognitive performance was worse at higher calendar age (p<0.001, all except Stroop test part 1). The offspring performed better compared to their partners on trial 3 (p = 0.005), the immediate (p = 0.016) and delayed (p = 0.004) recall of the 15-Picture Learning Test as well as on the interference and combined interference score of the Stroop test (p = 0.014 and p = 0.036, respectively) in the fully adjusted model. The difference between offspring and partners was estimated to be more than three years according to the observed difference in calendar age.

**Conclusions:**

Offspring of nonagenarian siblings with a familial history of longevity have better cognitive performance compared to the group of their partners of comparable age. This effect is independent of age-related diseases and known possible confounders. Possible explanations might be differences in subclinical vascular pathology between both groups.

## Introduction

Decline in cognitive performance is one of the most striking characteristics of the ageing process, already evident in middle age [1]–[3] and for some cognitive domains, like processing speed and spatial visualization, even at the age of 20 to 30 years [4]. With increasing life expectancy, the number of elderly people with severe cognitive impairment will grow rapidly, leading to a high demand on our health care. Understanding of decline in cognitive performance will therefore be one of the challenges of this century in order to be able to develop interventions focused on maintenance of cognitive performance with age.

Several causes and risk factors of decline in cognitive performance have been identified. Vascular pathology as well as cardiovascular risk factors, like alcohol use, smoking, hypertension and diabetes mellitus have been shown to play a prominent role in the development of cognitive decline [5]–[10]. Furthermore, decline in cognitive performance is associated with high systemic levels of inflammatory markers, such as C-reactive protein (CRP) and interleukin-6 (IL-6) [8], [11], [12]. Low socioeconomic status (including education, occupation and financial conditions) has been reported as predictor of cognitive decline [13]–[15]. The most important genetic risk factor for decline in cognitive performance is the *apolipoprotein E (APOE)* gene. Carriers of the *APOE ε4* allele are at an increased risk of dementia, whereas carriers of the *APOE ε2* allele might be protected [16], [17].

In the Leiden Longevity Study, we have previously demonstrated that middle-aged offspring of nonagenarian siblings with a familial history of longevity have a lower prevalence of age-related diseases, like myocardial infarction, hypertension and type II diabetes, compared to their partners sharing the same environmental conditions [18]. Major indicators of lifestyle however, i.e. body mass index, current smoking and level of education, are not different between both groups [18]. These results suggest that the biological age, which means a person’s rate of ageing compared to their calendar age [19], [20], of the offspring of nonagenarian siblings is lower compared to their partners. Based on this assumption, we studied whether the offspring of nonagenarian siblings perform better on cognitive tests than their partners.

## Methods

### Ethics Statement

Ethics approval was provided through the Medical Ethical Committee of the Leiden University Medical Center. Written informed consent was obtained from all subjects.

### The Leiden Longevity Study

The Leiden Longevity Study is a longitudinal cohort consisting of 421 families of long-lived Caucasian siblings of Dutch descent together with their offspring and the partners thereof [21]. The partners of the offspring were included as controls being of comparable age and sharing the same socioeconomic and geographical background as the offspring. Families were recruited if at least two long-lived siblings were alive and fulfilled the age-criterion of 89 years for males and 91 years for females. Sex-specific age-criteria were used due to the higher life-expectancy of females compared to males [18], [21]. No selection criteria on health or demographic characteristics were applied. Recruitment took place between July 2002 and May 2006 and the families are followed up since that time. Cognitive performance was tested in a random subgroup of subjects (250 offspring and 250 partners thereof) during a visit at the research center between September 2009 and December 2010.

### Cognitive Performance

The primary outcome was cognitive performance, which was tested for different cognitive domains like memory function, attention and processing speed. Memory function was assessed by the 15-Picture Learning Test (15-PLT). Subjects were shown 15 pictures of well-known items and then asked to recall as many as possible. The test was repeated three consecutive times and after 20 minutes. Outcome parameters were the number of correct pictures after each trial and after 20 minutes (delayed recall). The total number of correct answers after three trials was defined as the immediate recall. Furthermore, the number of incorrect pictures was reported for each trial. Attention and processing speed were assessed by the Stroop test and the Digit Symbol Substitution Test (DSST). The Stroop test consisted of three parts in which the subject had to name 40 items shown on a card. In part 1, the subjects were instructed to read color words, which were printed in black on card 1. In part 2, the card contained colored blocks and the subjects were asked to name the printed colors. In part 3, the card again contained color words, however printed in a discongruous ink color. The subjects were asked to name the ink color of the words. During all parts, the subjects were encouraged to read the card as fast as possible. The time needed to process each of the different parts as well as the errors during each trial were used as outcome parameters. Furthermore, the interfering effect of words upon the naming of colors (interference score) was assessed by calculating the difference in time needed for part 3 and 2 [22]. A combined interference score was calculated, in which both outcome parameters, time and number of errors, were combined. For each uncorrected error twice the average time per word for reading the card on which the error was made was added to the time needed to finish the card [22]. In the DSST, digits were presented and the subjects were asked to write the corresponding symbols in a blank space according to a given key. Outcome parameter was the number of correct digit-symbol combinations within 90 seconds. The DSST was added to the protocol later resulting in available data for 446 of the 500 subjects (223 offspring and 223 partners).

### Potential Confounders

Total number of years of education was calculated for each subject, based on self-reported information about the highest completed level of education. Conversion from highest educational level to total number of years of education was based on the Dutch educational system. Information on medical history was requested from the subjects’ treating physician including diabetes mellitus and cardiovascular diseases, defined as myocardial infarction, stroke and hypertension [18]. Information was obtained from 440 of the 500 subjects from the treating physicians. Questionnaires were used to obtain information about alcohol use and smoking. Inflammatory markers, i.e. high-sensitivity (hs) CRP and IL-6, were available in non-fasting serum samples for 480 of the 500 subjects at baseline. In 20 subjects non-fasting serum samples were not available due to technical problems or refusal of the subject. For hsCRP, the Hitachi Modular P 800 from Roche, Almere, the Netherlands was used [23]. IL-6 levels were determined with the Pelikine Compact human IL-6 ELISA kit from Sanquin reagents, Amsterdam, the Netherlands. *APOE* genotypes were determined within a genome-wide association study [24]. Three groups were defined for the statistical analysis including homozygotes of the *APOE ε3* allele (*ε3ε3*), carriers of the *APOE ε2* allele (*ε2ε2* and *ε2ε3*) and carriers of the *APOE ε4* allele (*ε4ε4*, *ε3ε4*, *ε2ε4*).

### Statistical Analysis

A cross-sectional analysis was performed to assess the association of cognitive performance with calendar age as well as with familial longevity in 250 offspring and 250 partners of the Leiden Longevity Study.

First, the association between calendar age and cognitive performance was assessed using linear regression analysis. Two different models were applied. In model 1, the analysis was adjusted for gender and years of education. Model 2 was as model 1 with further adjustments for comorbidities, alcohol use, smoking, inflammatory markers and *APOE* genotype. Subjects with hsCRP levels higher than 30 mg/L (n = 4) or IL-6 levels higher than 10 pg/mL (n = 2) were excluded from the analysis in model 2 in order to exclude possible influences of acute inflammatory conditions. Logistic regression was applied to assess the association between calendar age and the dichotomized number of mistakes reported for the 15-PLT and the Stroop test, i.e. one group with subjects having no mistakes and one group with subjects having one or more mistakes. The same two models described above were used for the logistic regression analysis.

Second, linear regression analysis was used to investigate the association between familial longevity (offspring versus partner status) and cognitive performance. Logistic regression analysis was used for the association between familial longevity and the two groups of subjects with and without mistakes for the 15-PLT and Stroop test. Again, two different models were applied. Model 1 included age, gender and years of education. Model 2 was as model 1 with further adjustments for comorbidities, alcohol use, smoking, inflammatory markers and *APOE* genotype. All p-values for differences between offspring and partners were adjusted for familial relationships among the offspring using robust standard errors.

Finally, a sensitivity analysis was performed excluding the subjects with the *APOE ε2ε4* genotype for both the analysis with calendar age and familial longevity. This subgroup was excluded because they have both the allele for an increased and decreased risk of dementia and could therefore attenuate the influence of *APOE* genotype on the results. Furthermore, the difference in cognitive performance between offspring and partners was expressed in years according to calendar age. For this calculation, the difference in cognitive performance between offspring and partners was divided by the difference in cognitive performance with calendar age per year of the fully adjusted model.

All statistical analyses were performed with Stata (version 12.0 for Windows, USA) and SPSS (version 20.0 for Windows, USA). P-values <0.05 were considered statistically significant.

## Results

Table 1 shows the characteristics of the study population. Offspring and partners had similar age and years of education. The prevalence of age-related diseases, like diabetes mellitus and hypertension, was lower among the offspring compared to their partners.

**Table 1:** Characteristics of the subjects stratified by offspring of nonagenarian siblings and their partners.

	Offspring	Partners
Characteristics	N = 250	N = 250
Demographics		
Females, n (%)	114 (45.6)	139 (55.6)
Age, years	66.3 (6.1)	65.7 (7.2)
Years of education, median (IQR)	12 (10–15)	12 (10–15)
Anthropometrics		
Height, cm	172.5 (8.9)	171.6 (8.8)
Weight, kg	78.7 (13.4)	79.2 (13.7)
Comorbidities*, n (%)		
Diabetes mellitus	7 (3.2)	20 (9.1)
Myocardial infarction	4 (1.8)	8 (3.6)
Stroke	5 (2.2)	5 (2.3)
Hypertension	56 (25.7)	66 (30.6)
COPD	13 (5.9)	9 (4.1)
Malignancy	13 (5.9)	21 (9.6)
Rheumatoid arthritis	2 (0.9)	3 (1.4)
Intoxications, n (%)		
Users of alcohol†	192 (78.0)	194 (78.5)
Former and/or current smokers	158 (64.2)	185 (74.9)
Inflammatory markers‡, median (IQR)		
hsCRP, mg/L	1.2 (0.65–2.49)	1.4 (0.71–2.87)
IL-6, pg/mL	0.30 (0.11–0.65)	0.36 (0.13–0.62)
*APOE* genotype, n (%)		
* ε2ε2*	2 (0.8)	1 (0.4)
* ε2ε3*	27 (11.1)	21 (8.4)
* ε2ε4*	11 (4.5)	10 (4.0)
* ε3ε3*	151 (60.4)	166 (66.7)
* ε3ε4*	51 (20.4)	47 (18.9)
* ε4ε4*	2 (0.8)	4 (1.6)

Values are expressed as mean (standard deviation), unless otherwise indicated. Abbreviations: IQR, interquartile range; COPD, chronic obstructive pulmonary disease; hsCRP, high-sensitivity C-reactive protein; IL-6, interleukin-6; *APOE*, *apolipoprotein E*.

* n = 220 for offspring and n = 220 for partners.

† Using ≥1 units per week.

‡ n = 240 for offspring and n = 240 for partners.

Table 2 and 3 present the association between calendar age and cognitive performance. Overall, higher calendar age was associated with worse cognitive performance. The association between calendar age and cognitive performance remained statistically significant after adjustment for known possible confounders, except for the Stroop test part 1 (table 2). The number of subjects with mistakes was higher at higher calendar age for the Stroop test part 3. No association with calendar age was found for the Stroop test part 2 and the 15-PLT (table 3). Sensitivity analysis excluding the subjects with the *APOE ε2ε4* genotype did not change the results.

**Table 2:** Cognitive performance dependent on calendar age in years.

	All subjects*	Model 1			Model 2		
Cognitive performance tests	N = 500	β	95% CI	p	β	95% CI	p
15-PLT, correct pictures							
trial 1	7.4 (0.08)	−0.06	−0.08, −0.04	<0.001	−0.07	−0.10, −0.04	<0.001
trial 2	10.4 (0.09)	−0.09	−0.12, −0.06	<0.001	−0.11	−0.14, −0.08	<0.001
trial 3	12.0 (0.09)	−0.08	−0.10, −0.06	<0.001	−0.08	−0.11, −0.06	<0.001
immediate recall	29.7 (0.23)	−0.23	−0.29, −0.17	<0.001	−0.26	−0.33, −0.19	<0.001
delayed recall	11.2 (0.10)	−0.07	−0.10, −0.05	<0.001	−0.08	−0.11, −0.05	<0.001
Stroop test, seconds							
part 1	20.3 (0.26)	0.08	0.01, 0.16	0.036	0.08	−0.01, 0.16	0.081
part 2	24.8 (0.23)	0.18	0.11, 0.24	<0.001	0.18	0.10, 0.25	<0.001
part 3	49.4 (0.65)	0.83	0.65, 1.00	<0.001	0.89	0.69, 1.08	<0.001
interference score	24.6 (0.56)	0.65	0.50, 0.81	<0.001	0.71	0.54, 0.89	<0.001
combined interference score	26.3 (0.69)	0.79	0.60, 0.98	<0.001	0.85	0.64, 1.07	<0.001
DSST†, correct answers	46.2 (0.51)	−0.71	−0.84, −0.58	<0.001	−0.67	−0.82, −0.52	<0.001

* Values are expressed as mean (standard error).

† N = 446. Abbreviations: β, estimate; CI, confidence interval; 15-PLT, 15-Picture Learning Test; DSST, Digit Symbol Substitution Test. Model 1: adjusted for gender and years of education. Model 2: as model 1+ diabetes mellitus, cardiovascular diseases (myocardial infarction, stroke and hypertension), alcohol use, smoking, high-sensitivity C-reactive protein, interleukin-6 and *apolipoprotein E* genotype.

**Table 3:** Cognitive performance expressed as number of subjects with mistakes dependent on calendar age in years.

	All subjects*	Model 1			Model 2		
Cognitive performance tests	N = 500	OR	95% CI	p	OR	95% CI	p
15-PLT†							
trial 1	28 (5.6)	1.00	0.95, 1.07	0.90	1.01	0.94, 1.09	0.74
trial 2	28 (5.6)	1.00	0.94, 1.06	0.88	1.01	0.94, 1.08	0.76
trial 3	20 (4.0)	0.99	0.92, 1.06	0.78	1.00	0.92, 1.09	0.97
immediate recall	47 (9.4)	1.00	0.96, 1.05	0.89	1.01	0.96, 1.07	0.71
delayed recall	35 (7.1)	0.98	0.93, 1.04	0.50	1.00	0.94, 1.06	0.96
Stroop test†							
part 2	36 (7.2)	1.05	0.99, 1.11	0.081	1.04	0.98, 1.11	0.22
part 3	134 (27.0)	1.09	1.05, 1.13	<0.001	1.10	1.06, 1.15	<0.001

* Values are expressed as number (%).

† Subjects with no mistakes = 0, subjects with one or more mistakes = 1. Abbreviations: OR, odds ratio; CI, confidence interval; 15-PLT, 15-Picture Learning Test. Model 1: adjusted for gender and years of education. Model 2: as model 1+ diabetes mellitus, cardiovascular diseases (myocardial infarction, stroke and hypertension), alcohol use, smoking, high-sensitivity C-reactive protein, interleukin-6 and *apolipoprotein E* genotype.

Table 4 and 5 show the association of familial longevity and cognitive performance, comparing offspring of nonagenarian siblings with a familial history of longevity with their partners. In the fully adjusted model, the offspring performed better compared to their partners on part 3, the immediate and delayed recall of the 15-PLT as well as on the (combined) interference score of the Stroop test (table 4). The number of subjects with mistakes reported for the 15-PLT was not different between offspring and partners. Among the offspring, the number of subjects with mistakes was lower for the Stroop test part 2 and 3 compared to their partners. After adjustment for possible confounders, the association remained statistically significant for the Stroop test part 3 (table 5).

**Table 4:** Cognitive performance dependent on familial longevity (offspring versus partner status*).

	Offspring†	Partners†	Model 1			Model 2		
Cognitive performance tests	N = 250	N = 250	β	95% CI	p	β	95% CI	p
15-PLT, correct pictures								
trial 1	7.4 (0.11)	7.4 (0.12)	−0.13	−0.44, 0.18	0.40	−0.28	−0.63, 0.07	0.11
trial 2	10.4 (0.13)	10.3 (0.14)	−0.23	−0.56, 0.10	0.17	−0.30	−0.67, 0.07	0.11
trial 3	12.1 (0.12)	11.9 (0.12)	−0.32	−0.63, −0.01	0.042	−0.47	−0.80, −0.15	0.005
immediate recall	29.9 (0.31)	29.6 (0.33)	−0.69	−1.48, 0.10	0.088	−1.06	−1.92, −0.19	0.016
delayed recall	11.3 (0.14)	11.1 (0.14)	−0.42	−0.77, −0.07	0.020	−0.58	−0.98, −0.18	0.004
Stroop test, seconds								
part 1	20.9 (0.38)	19.7 (0.34)	−1.27	−2.31, −0.23	0.017	−0.91	−2.13, 0.32	0.15
part 2	25.3 (0.33)	24.4 (0.30)	−0.86	−1.74, 0.02	0.057	−0.75	−1.77, 0.27	0.15
part 3	48.8 (0.86)	50.0 (0.97)	1.62	−0.67, 3.91	0.17	2.02	−0.56, 4.61	0.13
interference score	23.5 (0.74)	25.7 (0.84)	2.52	0.50, 4.54	0.015	2.84	0.57, 5.10	0.014
combined interference score	25.1 (0.92)	27.5 (1.02)	2.80	0.30, 5.30	0.028	3.12	0.21, 6.03	0.036
DSST‡, correct answers	46.3 (0.71)	46.2 (0.74)	−0.32	−2.16, 1.52	0.73	−0.82	−2.86, 1.21	0.43

* Offspring = 0, partner = 1.

† Values are expressed as mean (standard error).

‡ n = 223 for offspring and n = 223 for partners. Abbreviations: β, estimate; CI, confidence interval; 15-PLT, 15-Picture Learning Test; DSST, Digit Symbol Substitution Test. Model 1: adjusted for age, gender and years of education. Model 2: as model 1+ diabetes mellitus, cardiovascular diseases (myocardial infarction, stroke and hypertension), alcohol use, smoking, high-sensitivity C-reactive protein, interleukin-6 and *apolipoprotein E* genotype. Robust standard errors were used to account for familial relationships among the offspring.

**Table 5:** Cognitive performance expressed as number of subjects with mistakes dependent on familial longevity (offspring versus partner status*).

	Offspring†	Partners†	Model 1			Model 2		
Cognitive performance tests	N = 250	N = 250	OR	95% CI	p	OR	95% CI	p
15-PLT‡								
trial 1	13 (5.2)	15 (6.0)	1.13	0.53, 2.44	0.75	1.33	0.48, 3.68	0.58
trial 2	15 (6.0)	13 (5.2)	0.89	0.41, 1.94	0.77	0.95	0.37, 2.45	0.92
trial 3	10 (4.0)	10 (4.0)	1.09	0.44, 2.71	0.85	0.81	0.29, 2.28	0.69
immediate recall	24 (9.6)	23 (9.2)	0.94	0.51, 1.73	0.85	0.88	0.43, 1.82	0.74
delayed recall	16 (6.4)	19 (7.7)	1.32	0.63, 2.76	0.47	1.28	0.55, 2.99	0.57
Stroop test‡								
part 2	12 (4.8)	24 (9.6)	2.10	1.02, 4.32	0.043	2.03	0.86, 4.82	0.11
part 3	56 (22.6)	78 (31.3)	1.64	1.08, 2.50	0.021	1.69	1.05, 2.70	0.029

* Offspring = 0, partner = 1.

† Values are expressed as number (%).

‡ Subjects with no mistakes = 0, subjects with one or more mistakes = 1. Abbreviations: OR, odds ratio; CI, confidence interval; 15-PLT, 15-Picture Learning Test. Model 1: adjusted for age, gender and years of education. Model 2: as model 1+ diabetes mellitus, cardiovascular diseases (myocardial infarction, stroke and hypertension), alcohol use, smoking, high-sensitivity C-reactive protein, interleukin-6 and *apolipoprotein E* genotype. Robust standard errors were used to account for familial relationships among the offspring.

Sensitivity analysis excluding the subjects with the *APOE ε2ε4* genotype did not change the results.

**Figure 1:**
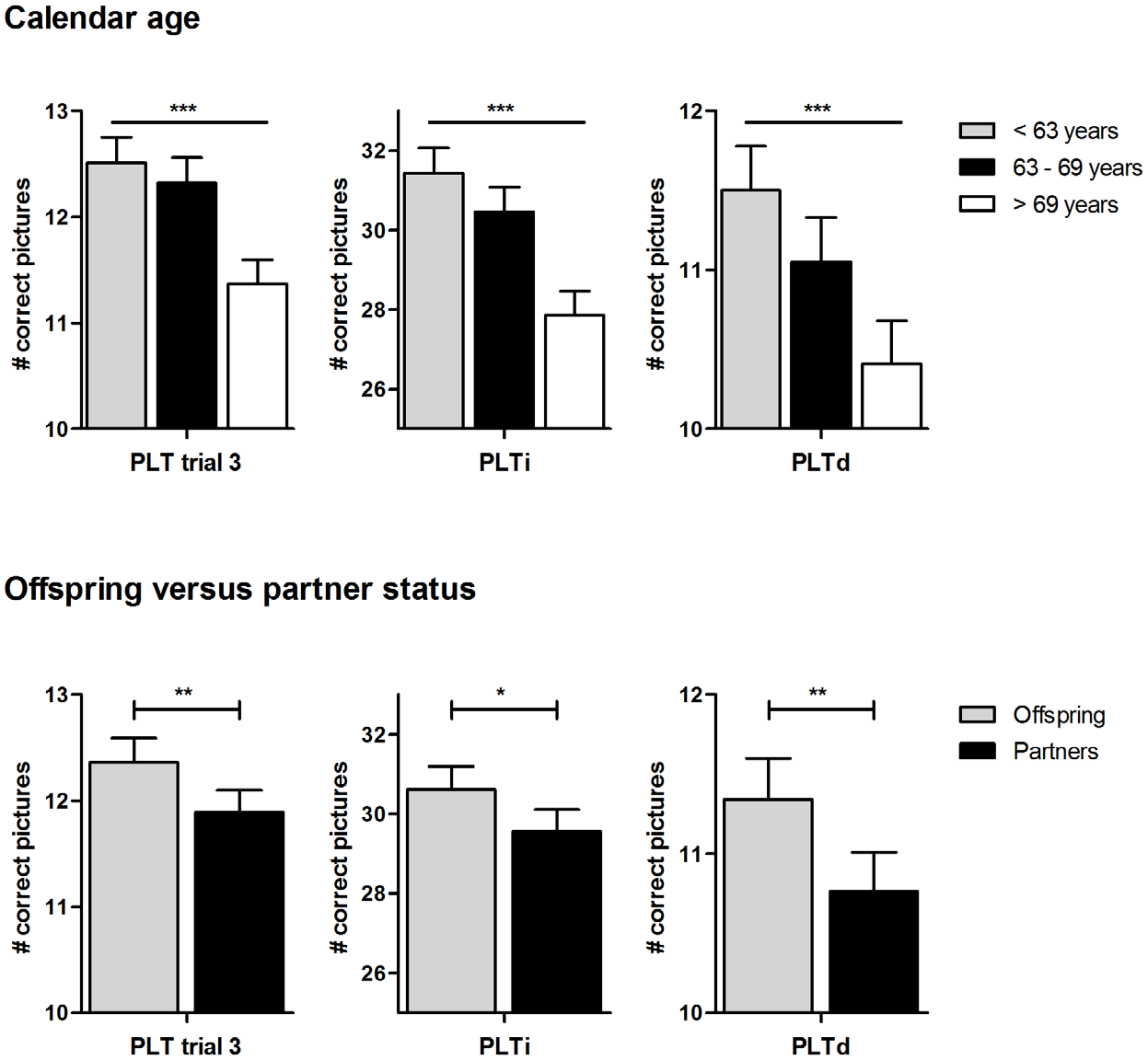
Association of the 15-Picture Learning Test with calendar age and familial longevity. In the upper and lower panel, estimated mean values for trial 3, the immediate (PLTi) and delayed (PLTd) recall of the 15-Picture Learning Test are shown in tertiles of calendar age and for offspring versus partner status, respectively. Error bars indicate standard error. Analyses on calendar age are adjusted for gender, years of education, diabetes mellitus, cardiovascular diseases, alcohol use, smoking, high-sensitivity C-reactive protein, interleukin-6 and *apolipoprotein E* genotype. P-values indicate p for trend. Analyses on offspring versus partner status are adjusted additionally for age and for familial relationships among the offspring using robust standard errors. P-values indicate the difference between offspring and partners. ***:p<0.001, **:p<0.01, *p<0.05.

**Figure 2:**
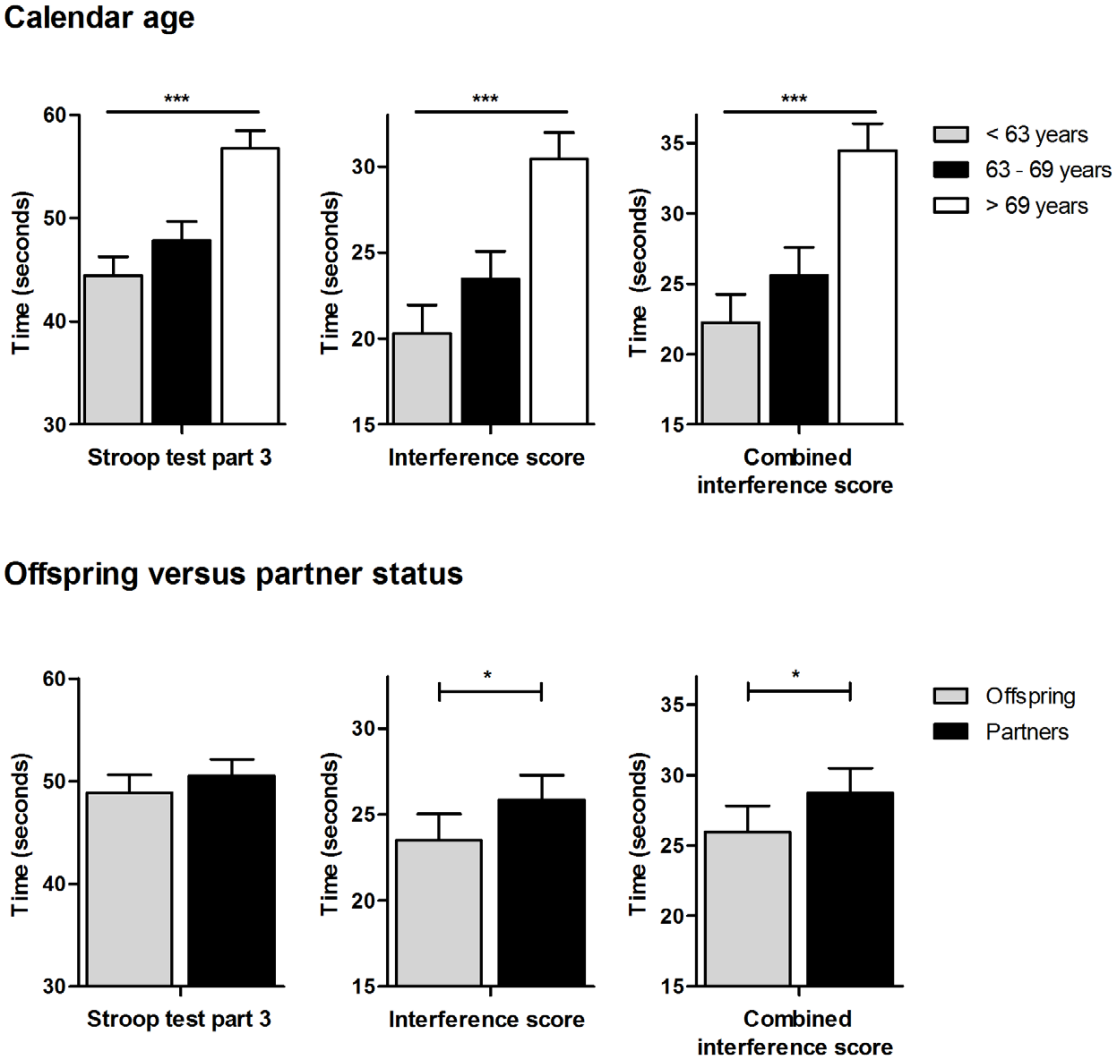
Association of the Stroop test with calendar age and familial longevity. In the upper and lower panel, estimated mean values for part 3, the interference score and combined interference score of the Stroop test are shown in tertiles of calendar age and for offspring versus partner status, respectively. Error bars indicate standard error. Analyses on calendar age are adjusted for gender, years of education, diabetes mellitus, cardiovascular diseases, alcohol use, smoking, high-sensitivity C-reactive protein, interleukin-6 and *apolipoprotein E* genotype. P-values indicate p for trend. Analyses on offspring versus partner status are adjusted additionally for age and for familial relationships among the offspring using robust standard errors. P-values indicate the difference between offspring and partners. Take note that lower Stroop scores indicate better cognitive performance.***:p<0.001, *:p<0.05.

Figure 1 and 2 show the estimated mean values of the 15-PLT and Stroop test, respectively, stratified for tertiles of calendar age (upper panel) and stratified for offspring and partners (lower panel). Preservation of cognitive performance of offspring compared to partners was estimated to be more than three years based on the difference in cognitive performance with calendar age per year in trial 3, the immediate and delayed recall of the 15-PLT as well as in the (combined) interference score of the Stroop test. The delayed recall of the 15-PLT showed the largest difference, with an estimated preservation of cognitive performance of the offspring of more than seven years.

## Discussion

The aim of this study was to compare cognitive performance between middle-aged offspring of nonagenarian siblings with a familial history of longevity and the group of their partners of comparable age and sharing the same environmental conditions. Cognitive performance was better among the offspring compared to their partners, even after adjustment for known possible confounders. Furthermore, higher calendar age was associated with worse cognitive performance. According to calendar age, the preservation of cognitive performance of the offspring compared to their partners was estimated to be more than three years.

Despite the relative small age range and relatively good health status of the subjects of the Leiden Longevity Study, a significant association between calendar age and cognitive performance was found. Adjustment for possible risk factors for cognitive decline, such as diabetes mellitus, cardiovascular diseases, alcohol use, smoking, systemic inflammation and *APOE* genotype did not change the results. The association between calendar age and cognitive performance was found in all cognitive domains that were tested, i.e. memory function, attention and processing speed. This makes it likely that processes during the ageing course play a prominent role in the development of decline in cognitive performance even from middle age [1]–[3].

Cognitive performance was better among the offspring of nonagenarian siblings compared to their partners, with whom they share their life. The difference in cognitive performance between offspring and partners remained statistically significant after adjustment for possible confounders [8]–[10]. This indicates that the difference in cognitive performance between offspring and partners cannot be accounted for by diabetes mellitus, cardiovascular diseases, alcohol use and smoking. Neither changed the results after adjustment for inflammatory markers, of which high systemic levels have been reported to be associated with decline in cognitive performance as well [8], [11], [12]. Furthermore, the association of cognitive performance with familial longevity remained statistically significant after adjustment for *APOE* genotype. The *APOE* genotype is besides one of the most important genetic risk factors for cognitive decline [16], [17], consistently shown to be associated with survival and longevity [25], [26]. A genome wide association study performed in the nonagenarian participants of the Leiden Longevity Study identified the *APOE ε4* isoform as deleterious to longevity, which was confirmed in a meta-analysis of three different replication cohorts [24]. Adjustment for familial relationships among the offspring did not change the results either, which excludes the influence of familial resemblance on the difference between offspring and partners in cognitive performance.

Altogether, the independence of the difference in cognitive performance between offspring and partners of above mentioned risk factors, suggests that the results have to be explained by other factors. One possible explanation is that the offspring are biologically younger compared to their partners, which means that the person’s rate of ageing of the offspring is slower compared to their partners. Based on the effect sizes of the association of cognitive performance with calendar age and familial longevity, the preservation of cognitive performance of the offspring was estimated to be more than three years according to calendar age. This finding of the offspring being biologically younger compared to their partners is in line with several other observations. The younger biological age of the offspring is reflected by their lower mortality rate, beneficial glucose and lipid metabolism, preservation of insulin sensitivity, preservation of naïve T-cell pool and resistance to cellular stress [18], [21], [27]–[29].

Another possible explanation is that the offspring have a better health status compared to their partners due to a more favorable development in utero or during early childhood. This explanation might be supported by the fact that the differences between offspring and partners in cognitive performance are already visible at middle age, when decline in cognitive performance is relatively small. However, data to test this possible explanation are currently not available and would require a familial multigenerational design.

Very recently we found differences in subclinical vascular pathology between offspring and partners. Assessment of magnetic resonance imaging scans in a subgroup of offspring and partners showed that the offspring had a lower periventricular as well as subcortical white matter load and a lower prevalence of lucunar infarcts compared to their partners [30]. Further research on the relation between the differences in subclinical vascular pathology and cognitive performance among the offspring and their partners is needed to get more insight into this possible causal pathway.

One of the strengths of our study is the unique study design of comparing middle-aged individuals, who are enriched for familial factors of longevity, to their partners. This gives the possibility to get more insight into determinants of healthy longevity. By including couples, the influence of socioeconomic status was relatively low making the groups highly comparable. The relative young age of the subjects is both a strength and limitation of the study. Differences in cognitive performance with calendar age in this relatively young study population were already observable; however, differences between offspring and partners may therefore be underestimated. Another limitation is the cross-sectional design of the present analysis, as cognitive performance data became available just recently.

In conclusion, offspring of nonagenarian siblings with a familial history of longevity showed a better cognitive performance compared to their partners being independent of known possible confounders. This makes it likely that cognitive performance is preserved with familial longevity. Further research on the possible causes of the relation between cognitive performance and familial longevity is needed in order to be able to get a better understanding of preservation of cognitive performance with age.
